# Rates of primary and secondary treatments for patients on active surveillance for localized prostate cancer—A population‐based cohort study

**DOI:** 10.1002/cam4.3341

**Published:** 2020-08-05

**Authors:** Rano Matta, Amanda E. Hird, Erind Dvorani, Refik Saskin, Gregory J. Nason, Girish Kulkarni, Ronald T. Kodama, Sender Herschorn, Robert K. Nam

**Affiliations:** ^1^ Division of Urology Sunnybrook Health Sciences Centre University of Toronto Toronto ON Canada; ^2^ Institute of Health Policy, Management and Evaluation University of Toronto Toronto ON Canada; ^3^ Institute of Clinical Evaluative Sciences University of Toronto Toronto ON Canada; ^4^ Division of Urology University Health Network University of Toronto Toronto ON Canada

**Keywords:** active surveillance, mortality, prostate cancer, prostatic neoplasms, recurrence

## Abstract

**Background:**

The rate of primary and secondary treatment while on active surveillance (AS) for localized prostate cancer at the general population level is unknown. Our objective was to determine the patterns of secondary treatments after primary surgery or radiation for patients who undergo AS.

**Methods:**

This was a population‐based retrospective cohort study of men aged 50‐80 years old in Ontario, Canada, between 2008 and 2016. We identified 26 742 patients with prostate cancer, a Gleason grade score ≤7, and an index prostate‐specific antigen ≤10 ng/mL. Patients were categorized as undergoing AS with or without delayed primary treatment (DT; treatment >6 months after diagnosis) versus immediate treatment (IT; treatment ≤6 months). Patients receiving DT and IT were propensity score matched and the rate of secondary treatment (surgery or radiation ± androgen deprivation treatment) was compared using Cox proportional hazards models.

**Results:**

We identified 10 214 patients who underwent AS and 11 884 patients who underwent IT. Among patients undergoing AS, 3724 (36.5%) eventually underwent DT and among them, 406 (10.9%) underwent secondary treatment. The median time to DT was 1.2 years (IQR 0.5‐8.1 years). The relative rate of undergoing secondary treatment was similar in the DT vs IT group (HR 0.92; 95% CI: 0.79‐1.08). The risk of death in the DT group was higher compared to patients who did not undergo treatment (HR 1.23, 95% CI: 1.01‐1.49).

**Conclusions:**

Among patients with localized prostate cancer on AS, one third undergo DT. The rate of secondary treatment was similar between the DT and IT groups. Patients in the DT group may experience a higher risk of mortality compared to those who remained on AS.

## Graphical abstract

For men with localized prostate cancer who are candidates for active surveillance, one third receive delayed primary treatment. Active surveillance is a safe approach, as there appears to be no adverse risk of progression with similar rates of secondary treatment compared to patients who underwent immediate treatment.

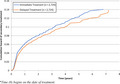

## 1 INTRODUCTION

Active surveillance (AS) for the management of patients with clinically localized prostate cancer is a recommended treatment option for patients with low‐risk disease.(1, 2, 3) A key benefit of AS is that patients can avoid unnecessary radical treatment, while only undergoing treatment when there is evidence of a change in status either based on tumor or host factors. The premise of undergoing treatment for patients while on AS is that patients are still candidates to undergo curative treatment with no adverse risk to their prognosis.

The reasons for patients to undergo primary surgical or radiation treatment while on AS can vary. Physicians may recommend treatment based on progression in prostate‐specific antigen (PSA) levels, histologic grade based on biopsy, or clinical stage based on clinical exam, although the clinical monitoring of patients undergoing AS is nonstandardized. Patients may also request treatment due to personal anxiety or other factors.(4) The rate of primary treatments while on AS ranges from 30% to 50% over 5 years.(5, 6) These rates are primarily reported from single and multi‐institutional series(5, 6, 7, 8) and clinical trials.(9, 10) The treatment rates among the general population are largely unknown. Furthermore, the rates of secondary treatments after surgery or radiation have not been well studied.

To better understand the outcomes associated with AS and delayed primary treatment, we evaluated the rates of primary and secondary therapy for patients undergoing treatment while on AS and compared their survival to control groups from a large population‐based cohort from Ontario, Canada.

## 2 METHODS

### 2.1 Data sources and setting

We conducted a population‐based, retrospective cohort study of all men ≥50 years and ≤80 years old who received a diagnosis of low‐ or intermediate‐risk prostate cancer in Ontario Canada with linked health administrative databases. In Ontario, all necessary healthcare services, physician services, and prescription medication information are recorded and held at the ICES (http://www.ices.on.ca). Each of the data sources used has been validated previously (Appendix S3). Our institutional Research Ethics Board approved the study protocol. All analyses were performed between June 2018 and January 2019 and were consistent with Strengthening the Reporting of Observational Studies in Epidemiology (STROBE) reporting guidelines (Appendix S4).

### 2.2 Study subjects

We identified all individuals with a new histologic diagnosis of Gleason 6 or 7 prostate cancer between April 1, 2008 and December 31, 2016, who received a prostate biopsy or transurethral resection of the prostate (TURP) within the preceding 3 months. We also required that their nearest PSA test result prior to or after their diagnostic biopsy/TURP (<180 days) was <10 ng/mL (index PSA). We excluded patients <50 or >80 years old to limit the population to those patients appropriate for AS. We also excluded patients with a Gleason score >7 and metastases ≤1 year following diagnosis. To establish the AS cohort, we excluded patients who received treatment ≤6 months after diagnosis (radical prostatectomy, external beam radiotherapy, brachytherapy, or ADT). To identify a comparison cohort of patients receiving immediate treatment (IT; treatment ≤6 months after diagnosis), we included all individuals with a new diagnosis of low or intermediate favorable‐risk prostate cancer who had received treatment with radical prostatectomy, external beam radiotherapy, brachytherapy, or ADT ≤6 months from diagnosis. Patients were followed until December 31, 2017 or censored if they died during follow‐up.

### 2.3 Exposure

The primary independent variable was the management strategy following prostate cancer diagnosis: AS with or without delayed primary treatment (DT; treatment >6 months after diagnosis) versus immediate treatment (IT; treatment ≤6 months) after the diagnosis of prostate cancer.

### 2.4 Covariates

We collected important patient and primary physician baseline characteristics that may confound the association between the initial management strategy following prostate cancer diagnosis and overall survival. Patient covariates were age at diagnosis, year of diagnosis, comorbidity score (Aggregate Diagnostic Groups [ADG] score), income quintile, geographic location (Local Health Integration Network [LHIN]), index PSA, and the number of prediagnosis prostate biopsies. We also captured a specific history of asthma or COPD, prior cancer diagnosis, diabetes, myocardial infarction (MI), congestive heart failure (CHF), cerebrovascular accident (CVA), and hypertension. The Gleason grade at the time of prostate cancer diagnosis was captured from the pathology staging information in the Ontario Cancer Registry, with only primary and secondary patterns available and without other information regarding biopsy core characteristics. For patients with missing Gleason grade information (n = 5665; 13%), we performed multiple imputation in SAS using Markov Chain Monte Carlo (MCMC) which assumes that all the variables in the imputation model have a joint multivariate normal distribution. We performed 10 iterations using all patient covariates described above without transformation. We then compared the baseline characteristics of the cohort derived from the imputation model to a cohort with complete cases only (ie, nonmissing Gleason score), and they were similar (not shown).

For the purposes of matching cohorts, we generated a propensity score (PS) using the following variables: age, year of diagnosis, ADG, income quintile, rurality, index PSA value, number of prediagnosis biopsies, history of asthma/COPD, prior cancer diagnosis, diabetes, myocardial infarction (MI), congestive heart failure (CHF), cerebrovascular accident (CVA), hypertension, and the Gleason score at the time of diagnosis.

### 2.5 Outcomes

The primary outcome was rate of secondary treatment (salvage radical prostatectomy, salvage radiation, or ADT for biochemical recurrence) after undergoing delayed primary treatment on AS. We also evaluated the predictors of receiving DT (radical prostatectomy, external beam radiotherapy ± ADT, brachytherapy ± ADT) while on AS. As a secondary outcome, we compared the risk of receiving salvage therapy among patients who received DT versus IT. We also compared overall mortality among those who received AS to matched nonprostate cancer controls from the general population of Ontario.

### 2.6 Analysis

We estimated the hazard of secondary treatments for patients receiving DT vs IT. Patients receiving DT and IT were matched 1:1 on PS, and index date (±6 months). We estimated the hazard of secondary treatment (salvage radiotherapy or prostatectomy) using Cox Proportional Hazards (CPH) models adjusting for patient and disease baseline characteristics that had a standard difference greater than 0.1 after matching. To account for the competing risks of death and treatment with ADT for biochemical recurrence, we used subdistributional hazards Fine‐and‐Gray models. We also evaluated in a separate CPH model, the hazard of receiving ADT for biochemical recurrence.

To evaluate the overall survival following AS with or without DT, we matched men undergoing AS to general population noncancer controls in a 1:1 ratio on age and comorbidity. We then evaluated the overall survival using a CPH, adjusting for treatment as a time‐varying covariate, and for patient baseline characteristics that had a standard difference greater than 0.1 after matching.

## 3 RESULTS

We identified 26 742 patients with a prostate cancer diagnosis by prostate needle core biopsy or TURP with a Gleason grade score of 7 or less, and an index PSA test result of ≤10 ng/mL. After applying exclusion criteria, 10 214 patients underwent AS (see Table S1) and 11 884 patients underwent IT within ≤6 months after the date of diagnosis (see Table S2).

Among patients diagnosed with prostate cancer (Table 1), the median age was 65 years (interquartile range [IQR] 60‐70 years), and patients in the AS group were older than the IT group (65.2 ± 7.3 years vs 64.5 ± 7.0 years; standard difference [SDiff] 0.09). Patients in the AS group had a higher level of comorbidity (mean ADG score 7.2 ± 3.2) compared to the patients in the IT group (6.8 ± 3.1; SDiff 0.11). Mean index PSA values were slightly higher in the AS group (5.30 ± 2.37 vs 5.02 ± 2.87 ng/mL; SDiff 0.1). Patients in the AS group had a higher proportion of Gleason score 6 cancer (n = 7252, 71%) compared to patients who had IT (n = 3727, 31.4%).

**Table 1:** Baseline characteristics of AS and IT cohorts, diagnosed with prostate cancer (Gleason ≤ 7) between April 1, 2008 and December 31, 2016

	Unmatched sample				Matched sample			
	IT, N = 11 884	AS, N = 10 214	Total, N = 22 098	Std diff	IT, N = 6085	AS, N = 6085	Total, N = 12 170	Std diff
Age (years)								
Median (IQR)	65 (59‐70)	65 (60‐70)	65 (60‐70)	0.08	65 (59‐70)	64 (59‐70)	64 (59‐70)	0.01
ADG								
0‐4	2908 (24.5%)	2310 (22.6%)	5218 (23.6%)	0.04	1446 (23.8%)	1489 (24.5%)	2935 (24.1%)	0.02
5‐9	6760 (56.9%)	5576 (54.6%)	12 336 (55.8%)	0.05	3457 (56.8%)	3392 (55.7%)	6849 (56.3%)	0.02
10‐14	2019 (17.0%)	2101 (20.6%)	4120 (18.6%)	0.09	1076 (17.7%)	1104 (18.1%)	2180 (17.9%)	0.01
15+	197 (1.7%)	227 (2.2%)	424 (1.9%)	0.04	106 (1.7%)	100 (1.6%)	206 (1.7%)	0.01
Income quintile								
Missing	40 (0.3%)	38 (0.4%)	78 (0.4%)	0.01	0	0	0	
1	1561 (13.1%)	1437 (14.1%)	2998 (13.6%)	0.03	835 (13.7%)	829 (13.6%)	1664 (13.7%)	0
2	2137 (18.0%)	1840 (18.0%)	3977 (18.0%)	0	1119 (18.4%)	1088 (17.9%)	2207 (18.1%)	0.01
3	2343 (19.7%)	2024 (19.8%)	4367 (19.8%)	0	1174 (19.3%)	1224 (20.1%)	2398 (19.7%)	0.02
4	2698 (22.7%)	2243 (22.0%)	4941 (22.4%)	0.02	1360 (22.4%)	1330 (21.9%)	2690 (22.1%)	0.01
5	3105 (26.1%)	2632 (25.8%)	5737 (26.0%)	0.01	1597 (26.2%)	1614 (26.5%)	3211 (26.4%)	0.01
Rural	1576 (13.3%)	1283 (12.6%)	2859 (12.9%)	0.02	763 (12.5%)	758 (12.5%)	1521 (12.5%)	0
Index PSA (ng/mL)								
Mean (95% CI)	5.02 ± 2.87	5.30 ± 2.37	5.15 ± 2.65	0.11	5.42 (5.35, 5.48)	5.45 (5.39, 5.51)	5.43 (5.39, 5.48)	0.02
0.00‐2.60	2513 (21.1%)	1519 (14.9%)	4032 (18.2%)	0.16	774 (12.7%)	847 (13.9%)	1621 (13.3%)	0.04
2.61‐4.00	864 (7.3%)	1155 (11.3%)	2019 (9.1%)	0.14	564 (9.3%)	568 (9.3%)	1132 (9.3%)	0
4.01‐9.99	8507 (71.6%)	7540 (73.8%)	16 047 (72.6%)	0.05	4747 (78.0%)	4670 (76.7%)	9417 (77.4%)	0.03
Prediagnostic biopsies (n)								
0	112 (0.9%)	1081 (10.6%)	1193 (5.4%)	0.42	112 (1.8%)	137 (2.3%)	249 (2.0%)	0.03
1	10 966 (92.3%)	8198 (80.3%)	19 164 (86.7%)	0.35	5453 (89.6%)	5450 (89.6%)	10 903 (89.6%)	0
2+	806 (6.8%)	935 (9.2%)	1741 (7.9%)	0.09	520 (8.5%)	498 (8.2%)	1018 (8.4%)	0.01
Gleason Score								
6	3727 (31.4%)	7252 (71.0%)	10 979 (49.7%)	0.86	3314 (54.5%)	3327 (54.7%)	6641 (54.6%)	0
7	8157 (68.6%)	2962 (29.0%)	11 119 (50.3%)	0.86	2771 (45.5%)	2758 (45.3%)	5529 (45.4%)	0

Among the patients undergoing AS, we identified 3724 (36%) patients who eventually received DT during the follow‐up period. The median time to DT from diagnosis was 1.2 years (IQR 0.5‐8.1 years). The most common form of treatment was radical prostatectomy (n = 2130; 21%) followed by radiotherapy (external beam or brachytherapy) (n = 1594; 16%). To examine the factors that were associated with receiving DT, we conducted a multivariable analysis accounting for competing hazard of death among patients undergoing AS (n = 10 214). The strongest factors were increasing PSA level (For PSA > 10, HR 7.8; 95% CI 6.5‐9.3), higher Gleason score (HR 2.3; 95% CI 2.1‐2.6), and increasing number of repeat biopsies (HR [per biopsy] 1.4; 95% CI 1.3‐1.5) (Table 2).

**Table 2:** Multivariable survival analysis examining the characteristics at diagnosis of receiving delayed treatment among patients on active surveillance (N = 10 214), accounting for competing hazard of death

	HR	95% CI	P‐value
PSA value (ref = 0.00‐2.60)			
(2.61‐4.00)	1.17	0.95‐1.444	0.1384
(4.01‐9.99)	2.28	1.94‐2.69	<0.0001
(10+)	7.74	6.38‐9.41	<0.0001
Age	0.97	0.96‐0.97	<0.0001
Total ADG	1.02	1.00‐1.03	0.0295
Number of biopsies prior to diagnosis (ref = 0)			
1	1.74	1.34‐2.26	<0.0001
2+	1.17	0.86‐1.59	0.3218
Postdiagnosis biopsies (per 1 increase)	1.37	1.26‐1.49	<0.0001
Gleason score (ref = 6)	2.34	2.11‐2.60	<0.0001

Note

Variables adjusted for but not shown in table: Income quintile, local health integration network (LHIN), and comorbidities (Asthma, COPD, prior cancer diagnosis, diabetes, myocardial infarction, congestive heart failure, cerebrovascular accident, and hypertension).

**Figure 1:**
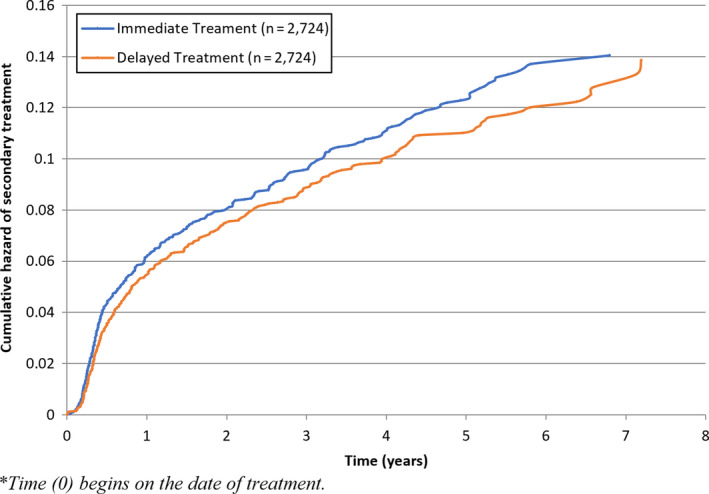
Cumulative hazard of secondary treatments based on initial management strategy for matched cohorts of patients receiving immediate treatment for localized prostate cancer versus delayed treatment while on active surveillance

To determine whether patients on AS who underwent DT had evidence of cancer progression, we examined rates of secondary treatments after undergoing delayed radical surgery or radiotherapy. Among the 2130 patients who had radical surgery on AS, 320 (15%) had subsequent radiation treatment and 40 (1.9%) had ADT for biochemical recurrence. Among the 1594 patients who had radiotherapy on AS, 14 (1%) had eventual salvage surgery and 32 (2%) had ADT (Table 3). When compared to patients who had immediate surgery or radiation (IT group), the rates for undergoing any secondary treatments were less in the patients who underwent treatment while on AS (Table 3). However, once we matched a subset of these patients by age, PS, and treatment date and after adjusting for competing risk of receiving ADT for biochemical recurrence and death, there was no difference in the risk of receiving secondary treatment between AS vs IT patients (HR 0.90; 95% CI 0.8‐1.1; *P* = .20) (Figure 1). There was also no difference in the risk of receiving ADT for biochemical recurrence (HR 1.1; 95% CI 0.8‐1.4; *P* = .67). To determine whether age could have biased the decision to undergo secondary treatments among the treated AS group, we restricted our analysis to patients ≤65 years. Among this subset, the rate of secondary treatments was significantly lower for patients in the DT group compared to the IT group (HR = 0.79; 95% CI: 0.7‐0.9; *P* = .02).

**Table 3:** Secondary treatments for patients undergoing delayed therapy on active surveillance or immediate treatment for localized prostate cancer

	Salvage/adjuvant RT	Salvage RP	ADT (biochemical recurrence)	Death	End of follow up, or loss OHIP eligibility
Delayed treatment (n = 3724)					
RT	0 (0.00%)	14 (0.88%)	32 (2.01%)	147 (9.22%)	1401 (87.89%)
RP	320 (15.02%)	0 (0.00%)	40 (1.88%)	43 (2.02%)	1727 (81.08%)
Immediate treatment (n = 11 586)					
RT	0 (0.00%)	25 (0.63%)	134 (3.36%)	226 (5.66%)	3606 (90.35%)
RP	1710 (22.51%)	0 (0.00%)	145 (1.91%)	119 (1.57%)	5621 (74.01%)

**Figure 2:**
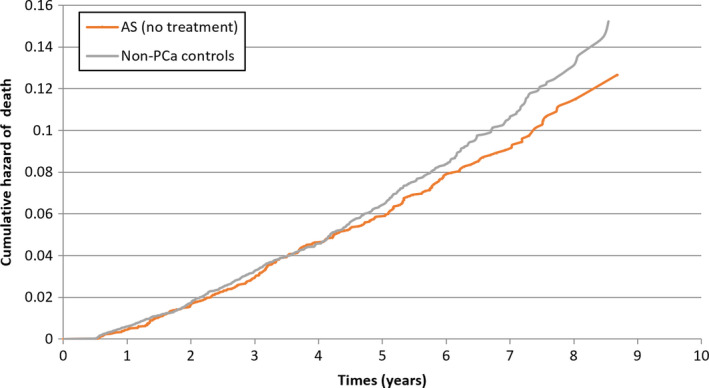
Cumulative hazard of death for active surveillance patients not receiving treatment and nonprostate cancer patients matched on age and comorbidities

To examine the natural history of patients on AS, we compared cumulative overall mortality rates between the AS group to noncancer controls from the general population. The number of patients with prostate cancer‐specific deaths was too few for analysis. We matched 7010 patients from the general population with no history of prostate cancer by age and comorbidity to 7010 patients undergoing AS. When compared to the noncancer population controls, the cumulative rate of death among patients on AS who never underwent treatment was similar (Figure 2), although there was a lower rate of mortality in the AS group (HR 0.76, 95% CI: 0.67‐0.86, *P* < .0001). Because of immortal time bias, we examined the effect of undergoing treatment while on AS using a time‐varying analysis. For patients who underwent DT, the cumulative rate of death was higher compared to patients who remained on AS, with an adjusted hazard ratio of 1.23 (95% CI: 1.01‐1.49, *P* = .03).

## 4 DISCUSSION

From a large population‐based cohort of 10 214 men who underwent AS, 36% of patients underwent delayed primary treatment and the rate of treatment was associated with increasing PSA, grade, and number of repeat biopsies. For those who undergo delayed primary treatment, there appears to be no adverse rates of progression given the same rates of secondary treatments between patients who undergo immediate or delayed treatment (adjusted HR 0.90, 95% CI 0.8‐1.1). When compared to the noncancer controls from the general population, the overall mortality rates were lower in the AS group. However, the adjusted overall mortality rates were higher among patients who underwent DT compared to patients who remained on AS.

To our knowledge, this is the first population‐based study to report the outcomes for patients with localized prostate undergoing AS cancer that examines rates of secondary treatment and overall survival with comparison to the general population. A major strength of this study includes population‐level data with the ability to follow patients after their diagnosis and primary treatment irrespective of where they were managed within the province.

Our rates of DT were lower compared to other studies. Bokhorst et al report on outcomes of the PRIAS study, including 5302 men across 18 countries. At 5 and 10 years of follow‐up, 52% and 73% of men, respectively, had discontinued AS.(8) Klotz et al in their large institutional cohort, the Sunnybrook Active Surveillance program, demonstrated that at 5, 10, and 15 years, 75.7%, 63.5%, and 55.0% of patients remained untreated and on surveillance, respectively.(5) Tosoian et al, in their large institutional cohort from Johns Hopkins, report that 50% of patients receive curative treatment at 10 years and 57% at 15 years.(7) With administrative data, we were unable to determine the reason for DT among patients undergoing AS.

The rates of secondary treatments (with salvage surgery, adjuvant/salvage radiation, or ADT) in the AS group were not higher than in patients who underwent IT. The rates of secondary treatment can provide a surrogate measure of prostate cancer progression. Thus, it is likely that patients did not experience any significant cancer progression based on these rates. This may suggest that the appropriate selection of patients to undergo primary treatment while on AS prevented patients from experiencing cancer progression, despite not having a standardized protocol for the follow‐up of these patients. Considering that these results may bias toward a better outcome for the AS group (since they are being compared to patients progressing on AS), this emphasizes the safety of AS as a management strategy.

The higher mortality rate observed for patients undergoing delayed primary treatment while undergoing AS is likely a reflection of the higher levels of comorbidity in the AS group and the burden of these comorbidities increasing over time while on AS. Nevertheless, the comparison to noncancer controls from the general population provides an important reference that this bias does not adversely affect patient outcome for patients undergoing AS. Indeed, the AS group eventually showed a lower mortality rate than noncancer controls likely due to surveillance bias (ie, their increased interaction with healthcare).

Relative to other AS cohorts, a strength of this study is the use of nonrestrictive eligibility criteria for AS to increase the generalizability of our findings. Previously, Komisarenko et al compared outcomes within their institutional AS database using more selective criteria and did not observe significant improvement in the relative risk of grade reclassification or biochemical failure after treatment.(11)

Another strength of this study that improves its generalizability includes the omission of a defined follow‐up protocol for AS. Follow‐up protocols for patients on AS differ depending on various studies. While all the cohorts monitor patients in some way using PSA, digital rectal exam (DRE), and repeat prostate biopsies, no study has defined the optimal surveillance protocol for AS. Klotz et al followed patients on AS with PSA every 3 months for 2 years then every 6 months, as well as prostate biopsy at 12 months, then every 3‐4 years. Men with a PSA doubling time of less than 3 years (before 2009) upgrading on biopsy and clinical progression were recommended to undergo definitive treatment.(5) Bokhorst et al (PRIAS study) followed patients with PSA measurements every 3 months for the first 2 years and PSA measurements every 6 months thereafter. Repeat prostate biopsies were scheduled after 1, 4, and 7 years; in case of a PSA doubling time (PSA‐DT) between 3 years and 10 years, annual repeat biopsies were advised.(8)

A general limitation of all studies using administrative databases is the potential for misclassification. Specifically, in this study, the administrative databases used do not specifically record the management strategy after prostate cancer diagnosis, and therefore the strategy was deduced based on eligibility criteria defined in the Methods section. Previously, Richard et al explored various eligibility criteria for AS using this same provincial database(12) and found that patient characteristics were similar regardless. Also, the absence of prediagnosis information on PSA density, clinical stage, family history, or urinary symptoms and postdiagnosis biopsy results are important limitations that may impact the decision to pursue IT or DT once on AS. As well we do not have pathological information such as core biopsy percentage involvement and number of cores, which limits our ability to further classify prostate cancer risk among the cohort (intermediate favorable vs unfavorable).

It remains unclear whether DT while on AS makes any impact on the natural history of prostate cancer. While our study shows that the selection of the patients to undergo DT while on AS appeared to be appropriate with rates of secondary treatment similar to patients who underwent IT, the overall mortality rates were higher given their higher level of comorbidities. Thus, since our patients who remained on AS did not have higher overall mortality rates than the general population, it is possible that DT would make no difference in outcome for patients on AS. Future research will be required to examine this possibility.

## 5 CONCLUSIONS

From a large population‐based cohort of men who underwent AS, one third of patients undergo primary treatment and the rate of treatment was associated with increasing PSA, grade, and number of repeat biopsies. AS as a management strategy appears safe as the risk of undergoing secondary treatments in the delayed treatment group was not significantly different when compared to patients who underwent immediate treatment for localized disease. However, patients in the delayed treatment group experience a higher risk of all‐cause mortality compared to those who remained on AS.

## DATA SHARING

The data that support the findings of this study are available from ICES and typically not shared due to privacy restrictions.

## CONFLICT OF INTEREST

All authors declare no conflicts of interest.

## AUTHOR CONTRIBUTIONS

Rano Matta: conceptualization, formal analysis, investigation, methodology, project administration, validation, writing – original draft, and writing – review and editing. Amanda Hird: investigation, methodology, project administration, and writing – review and editing. Erind Dvorani: data curation, formal analysis, investigation, methodology, project administration, validation, and writing – review and editing. Refik Saskin: formal analysis, investigation, methodology, project administration, supervision, validation, and writing – review and editing. Girish Kulkarni: investigation, methodology, and writing – review and editing. Ronald Kodama: investigation, methodology, and writing – review and editing. Sender Herschorn: investigation, methodology, and writing – review and editing. Robert Nam: conceptualization, formal analysis, funding acquisition, investigation, methodology, project administration, resources, supervision, validation, writing – original draft, and writing – review and editing.

## Supporting information

Supplementary Material
