# Supplementary material for: Rates of primary and secondary treatments for patients on active surveillance for localized prostate cancer—A population‐based cohort study
Source: Cancer Med. 2020 Aug 5;9(19):6946–53. doi: 10.1002/cam4.3341 (PMC7541139; doi:10.1002/cam4.3341)
Supplement: Supplementary file 1 — Supplementary Material [file CAM4-9-6946-s001.docx]

**Appendix Table 1 Study flow inclusion and exclusion criteria for AS cohort**

| Criteria | Frequency | Total cohort size |
| --- | --- | --- |
| PCa diagnosis (from April 1, 2008 to December, 31, 2016) with biopsy or TURP in prior 3 months, and closest PSA test <10 within 180 days of biopsy/TURP  AND  Did not receive treatment (prostatectomy, external radiotherapy, brachytherapy, ADT) within 6 months of diagnosis date | 11,455 |  |
| Patients younger than 50 or older than 80, females | 891 |  |
| Exclude patients with prior treatment or death before index | 76 |  |
| Patients with metastasis within 1-year following diagnosis | 71 |  |
| Gleason > 7 | 203 | **10,214** |

**Appendix Table 2 Study flow inclusion and exclusion criteria for IT cohort**

| Criteria | Frequency | Total cohort size |
| --- | --- | --- |
| PCa diagnosis (from April 1, 2008 to December, 31, 2016) with biopsy or TURP in prior 3 months, and closest PSA test <10 within 180 days of biopsy/TURP  AND  Received treatment (prostatectomy, external radiotherapy, brachytherapy, ADT) within 6 months of diagnosis | 15,287 |  |
| Patients younger than 50 or older than 80, females | 1,026 |  |
| Exclude patients with death before index | 7 |  |
| Patients with prior treatment (prostatectomy, external radiotherapy, brachytherapy, ADT) | 220 |  |
| Patients with metastasis within 1-year following diagnosis | 288 |  |
| Gleason > 7 | 1,862 | **11,884** |

**Appendix 3**

Using ICES as a data repository, we linked a number of administrative datasets including the Ontario Cancer Registry, a provincial registry which is more than 95% complete(13); the Canadian Institute for Health Information (CIHI) Discharge Abstract Database (DAD) which contains records for each hospitalization(14); the CIHI National Ambulatory Care Reporting System (NACRS) which contains records for ambulatory and emergency room visits; the Ontario Health Insurance Plan (OHIP) which tracks claims paid for physician billings, laboratories, and out-of-province providers (OHIP fee codes are provided for specific procedures with specific indications)(15); the Ontario Registrar General – Death database for vital status and cause of death information; and the Registered Persons (RPDB) for demographic information.

**Appendix 4 Strengthening the Reporting of Observational Studies in Epidemiology (STROBE) Checklist**

|  | Item No | Recommendation | Page  No |
| --- | --- | --- | --- |
| **Title and abstract** | 1 | (*a*) Indicate the study’s design with a commonly used term in the title or the abstract | 1 |
|  |  | (*b*) Provide in the abstract an informative and balanced summary of what was done and what was found | 3-4 |
| Introduction | | | |
| Background/rationale | 2 | Explain the scientific background and rationale for the investigation being reported | 5 |
| Objectives | 3 | State specific objectives, including any prespecified hypotheses | 5 |
| Methods | | | |
| Study design | 4 | Present key elements of study design early in the paper | 8-9 |
| Setting | 5 | Describe the setting, locations, and relevant dates, including periods of recruitment, exposure, follow-up, and data collection | 6 |
| Participants | 6 | (*a*) *Cohort study*—Give the eligibility criteria, and the sources and methods of selection of participants. Describe methods of follow-up  *Case-control study*—Give the eligibility criteria, and the sources and methods of case ascertainment and control selection. Give the rationale for the choice of cases and controls  *Cross-sectional study*—Give the eligibility criteria, and the sources and methods of selection of participants | 6, Supp 1-2 |
|  |  | (*b*) *Cohort study*—For matched studies, give matching criteria and number of exposed and unexposed  *Case-control study*—For matched studies, give matching criteria and the number of controls per case | NA |
| Variables | 7 | Clearly define all outcomes, exposures, predictors, potential confounders, and effect modifiers. Give diagnostic criteria, if applicable | 7 |
| Data sources/ measurement | 8* | For each variable of interest, give sources of data and details of methods of assessment (measurement). Describe comparability of assessment methods if there is more than one group | 6-7 |
| Bias | 9 | Describe any efforts to address potential sources of bias | 8 |
| Study size | 10 | Explain how the study size was arrived at | Supp 1-2 |
| Quantitative variables | 11 | Explain how quantitative variables were handled in the analyses. If applicable, describe which groupings were chosen and why | 7-8 |
| Statistical methods | 12 | (*a*) Describe all statistical methods, including those used to control for confounding | 8 |
|  |  | (*b*) Describe any methods used to examine subgroups and interactions | NA |
|  |  | (*c*) Explain how missing data were addressed | 7-8 |
|  |  | (*d*) *Cohort study*—If applicable, explain how loss to follow-up was addressed  *Case-control study*—If applicable, explain how matching of cases and controls was addressed  *Cross-sectional study*—If applicable, describe analytical methods taking account of sampling strategy | NA |
|  |  | (*e*) Describe any sensitivity analyses | NA |
| **Results** |  |  |  |
| Participants | 13* | (a) Report numbers of individuals at each stage of study—eg numbers potentially eligible, examined for eligibility, confirmed eligible, included in the study, completing follow-up, and analysed | 10 |
|  |  | (b) Give reasons for non-participation at each stage | NA |
|  |  | (c) Consider use of a flow diagram | NA |
| Descriptive data | 14* | (a) Give characteristics of study participants (eg demographic, clinical, social) and information on exposures and potential confounders | 10-11 |
|  |  | (b) Indicate number of participants with missing data for each variable of interest | NA |
|  |  | (c) *Cohort study*—Summarise follow-up time (eg, average and total amount) | 10 |
| Outcome data | 15* | *Cohort study*—Report numbers of outcome events or summary measures over time | 10-11 |
|  |  | *Case-control study—*Report numbers in each exposure category, or summary measures of exposure | NA |
|  |  | *Cross-sectional study—*Report numbers of outcome events or summary measures | NA |
| Main results | 16 | (*a*) Give unadjusted estimates and, if applicable, confounder-adjusted estimates and their precision (eg, 95% confidence interval). Make clear which confounders were adjusted for and why they were included | 10-11 |
|  |  | (*b*) Report category boundaries when continuous variables were categorized | NA |
|  |  | (*c*) If relevant, consider translating estimates of relative risk into absolute risk for a meaningful time period | NA |
| Other analyses | 17 | Report other analyses done—eg analyses of subgroups and interactions, and sensitivity analyses | 10-11 |
| **Discussion** |  |  |  |
| Key results | 18 | Summarise key results with reference to study objectives | 13 |
| Limitations | 19 | Discuss limitations of the study, taking into account sources of potential bias or imprecision. Discuss both direction and magnitude of any potential bias | 13-14 |
| Interpretation | 20 | Give a cautious overall interpretation of results considering objectives, limitations, multiplicity of analyses, results from similar studies, and other relevant evidence | 15 |
| Generalisability | 21 | Discuss the generalisability (external validity) of the study results | 14-15 |
| **Other information** |  |  |  |
| Funding | 22 | Give the source of funding and the role of the funders for the present study and, if applicable, for the original study on which the present article is based | 18 |
